# Geographical accessibility of medical resources, health status, and demand of integrated care for older people: a cross-sectional survey from Western China

**DOI:** 10.1186/s12877-024-04987-2

**Published:** 2024-05-20

**Authors:** Taoyu Lin, Wei Guo, Yuanyuan Li, Xiaoying Guo, Xue Bai, Rui Min

**Affiliations:** 1 The People’s Hospital of Suzhou New District, Suzhou, 215129 China; 2 Nursing Department, School of Medicine, Shihezi University, Xinjiang, 832008 China; 3 School of Nursing, Tongji Medical College, Huazhong University of Science and Technology, Wuhan, 430030 China; 4 School of Public Health, Tongji Medical College, Huazhong University of Science and Technology, Wuhan, 430030 China

**Keywords:** Integrated care for older people (ICOPE), Geographical accessibility of medical resources (GAMR), Health status, Propensity score matching (PSM)

## Abstract

**Background:**

The World Health Organization (WHO) published the Integrated Care for Older People (ICOPE) framework to help healthcare providers cope with the population aging crisis. However, the relevant evidence on the demands of older people and the compensatory capacity of the environment is limited. This study reports for the first time the level of the ICOPE demand in Western China that includes the impact of geographic accessibility of medical resources (GAMR) on ICOPE demand and the potential mechanism of health status.

**Methods:**

A cross-sectional questionnaire survey was conducted among 1200 adults aged 60 years and older selected through multi-stage stratified cluster sampling to obtain relevant data, including ICOPE demand, health status, and GAMR. Propensity score matching (PSM) was used to analyze the impact of GAMR on ICOPE demand among older people and those with different health statuses.

**Results:**

Among the prospective research participants, 1043 were eligible for the study. The mean score of ICOPE demand among all participants was 3.68 (standard deviation [*SD*] = 0.78). After adjusting for covariates between high and low GAMR groups (1:1 match), ICOPE demand was significantly higher in the low GAMR group than in the high GAMR group (average treatment effect on the treated [ATT] = 0.270, *p* < 0.05). For both good and poor self-rated health status, the ICOPE demand of the low GAMR group was significantly higher than that in the high GAMR group (ATT = 0.345, *p* < 0.05; ATT = 0.190, *p* < 0.05). For chronic diseases, the ICOPE demand of older people with multimorbidity in the low GAMR group was significantly higher than that in the high GAMR group (ATT = 0.318, *p* < 0.01).

**Conclusions:**

The older population in Western China has a relatively high demand for ICOPE. Low GAMR is a key factor in ICOPE demand growth in this region. It accelerates demand release for both older people with multimorbidity and self-perceptions of health.

**Supplementary Information:**

The online version contains supplementary material available at 10.1186/s12877-024-04987-2.

## Introduction

Declining fertility, reduced mortality rates, and longer life expectancy accelerate population aging worldwide. However, increased longevity is not always accompanied by prolonged good health [1–3]. Older people may experience persistent poor health due to chronic disease, multimorbidity (the coexistence of multiple chronic diseases), and geriatric syndromes (e.g., weakness, urinary incontinence, fall tendency). Confronted by these demographic changes, World Health Organization (WHO) is actively exploring the Integrated Care for Older People (ICOPE) approach, which aims to integrate health and social care services to establish a continuum of care model to prevent, slow, or reverse declines in the physical and mental capacities of older people [4, 5].

**Fig. 1:**
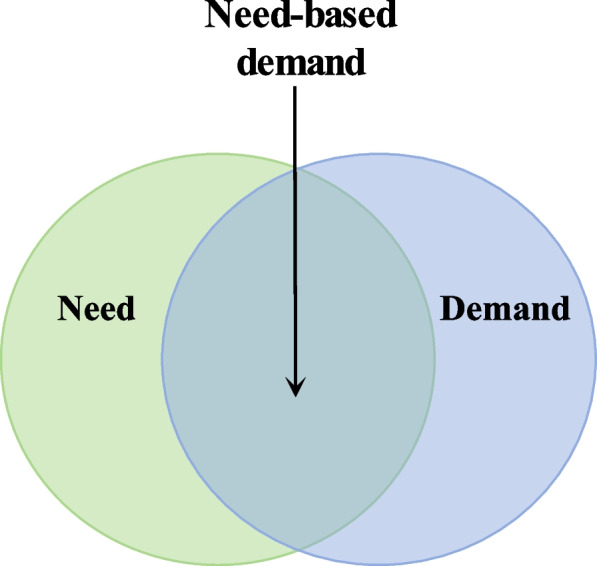
Need demand and need-based demand

The supply of ICOPE is limited by the size of the budget, the production technology, and the availability of scarce resources such as healthcare professionals, making it difficult to meet all the needs of older people [6]. It is urgent to identify and prioritize the priorities, needs, and preferences (i.e., need-based demands) of older people within the capacity of the government [4] that are affordable for individuals and governments (Fig. 1) [6, 7]. In this study, we define ICOPE demand as need-based demands considered a priority for health and social care services.

Existing evidence on ICOPE demand of older people has mostly derived from high-income countries, and demand types were mostly classified by service content. The main categories included systematic classification (health and social services) [8], the classification by health dimensions (physical, psychological, social) [9], and detailed and comprehensive classification (environmental, physical, psychological, social) [10, 11]. The WHO baseline report, the Decade of Healthy Ageing, shows that approximately 14% of older people globally could not meet the basic daily demands necessary to lead a meaningful and dignified life. This proportion may be higher in reality due to limited comparable data [5]. Furthermore, important information on older adults’ health status and function has often been overlooked in health demands statistics of older people [12]. WHO emphasizes different economic developments for countries to actively explore and accumulate experience to improve primary care applicability despite the lack of evidence on the demands of older people and the environmental ability to compensate for their health [13, 14].

Health is an important factor of ICOPE demand. Previous studies positively associate the extent of impaired health and the integrated demand of older people [8, 10, 15, 16]. The comprehensive evaluation of health status is generally measured by objective and subjective health statuses [17–20]. The former usually focuses on objective information regarding the physical dimensions of health (e.g., health measurement data, diagnosis of diseases), and the latter indicates an individual’s perception of their overall physical, mental, and social health and is often evaluated by self-reported health status [21]. Therefore, it is reasonable to assume that objective health status may affect the individual’s subjective health status and further impact the ICOPE demand. However, as an individual’s perception of health is not completely affected by objective health status [19, 22], evaluating ICOPE demand requires comprehensive access to health information for older people.

The diversity of health and social care demands is mainly caused by differences in individual characteristics and social-environmental factors. Individual characteristics include fixed characteristics, such as age and sex, in addition to those characteristics that have certain mobility or reflect social status, such as occupation, educational level, or income, which determine the equity of access to health resources [23]. Social–environmental factors include social networks, accessibility and affordability of medical resources, the built environment, and health and social policies [5]. The social network is a microenvironment close to the individual and is easily perceived and selected by individuals to provide emotional and instrumental support to older people, affecting their physical and mental health [24]. Accessibility and affordability of medical resources are largely out of the control or choice of an individual and are also the root cause of health inequalities [4, 5, 23, 25, 26].

Recently, while promoting a universal health coverage strategy, countries worldwide have been focusing on improving the proportion of the population and their economic burden of accessing medical services and have achieved initial results. However, these efforts are insufficient to improve the geographical accessibility of medical resources (GAMR) which includes the distance, transportation resources, and time gap between people and medical and health institutions [27]. Evidence shows that the older population is more vulnerable to the geographical inaccessibility of medical resources, thereby reducing the utilization of healthcare services and exacerbating declines in intrinsic and functional capacity [28]. Considering the close link between older adults’ health and demands, all factors that promote or hinder health affect health and potentially instigate changes in the demands of older people. Therefore, health organizations must consider this link when determining priorities to meet the ICOPE demand.

China is one of the pilot countries for implementing ICOPE [3], with the fastest aging population worldwide [4]. China’s aging population is mainly characterized by a high proportion of disabled and semi-disabled older people. Additionally, a large health gap exists between urban and rural areas and between regions, especially in western rural areas. By 2050, China’s older population will reach 449 million, of which 28.1% of adults aged 60 and older will be disabled or semi-disabled. Meanwhile, 53.2% of the rural population consists of older adults, higher than that of 22% in urban areas [29]. Similar to countries worldwide, China’s older population also exhibits health inequality. Especially in the western region with low economic levels, the inequality phenomenon is more prominent and shows a slow improvement trend [30]. Compared with the eastern and central regions in China, the population in the western region has a heavy economic burden, and low health management and health security levels, and older people face prominent problems such as a high dependency ratio, weak social support network, and low physical and mental health levels [31–33]. Significantly, this region had a vast territory and a small population, and the GAMR problem may be more prominent than other regions. A review of previous studies found a missing link of evidence between the GAMR and the health status and the ICOPE demand of local older people.

This study conducted a cross-sectional survey of older people in Western China and reports for the first time the level of ICOPE demand in Western China that includes the impact of GAMR on ICOPE demand and the underlying mechanism of health status. The study provides first-hand data on reducing health inequities in service supply in China and also provides quantifiable evidence for ICOPE demand analysis in countries with similar national conditions and population aging.

## Methods

### Study design and participants

This study selected Xinjiang Production and Construction Corps (XPCC) as the sample area. As mentioned in our previous study [34], the XPCC is located in Western China, an important part of the core area of China’s Silk Road Economic Belt. This region has a high degree of population aging and poor accessibility to medical resources [35].

**Fig. 2:**
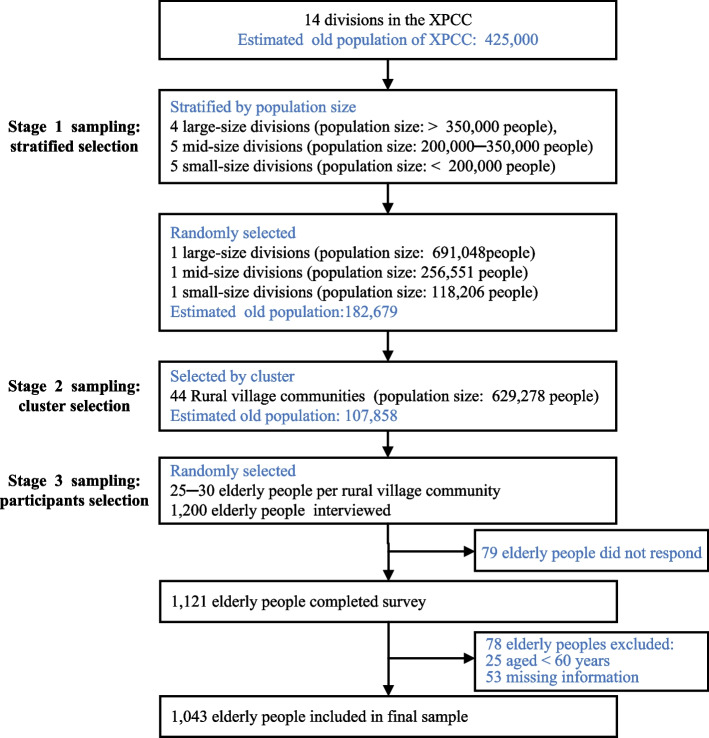
The sampling procedure

This study used a cross-sectional survey design and multistage, stratified, and cluster probability sampling methods to select participants aged 60 years and older from the XPCC. The predetermined sample size was 1200 participants, based on a prevalence of 54.5% of ICOPE demand among older people [36]. In Stage 1, according to the XPCC population at the end of 2020, the 14 divisions of the Corps were divided into three layers: four large-size divisions (population greater than 350,000), five mid-size divisions (population between 200,000 and 350,000), and five small-size divisions (population less than 200,000). One division was randomly (using random numbers generated by IBM® SPSS® Statistics software) selected from each layer. In Stage 2, rural village communities were selected by cluster sampling from each division. In Stage 3, 25–30 individuals aged 60 years or older were systematically randomly selected from each selected community (Fig. 2).

Eligible study participants were permanent older residents who had lived in their current residence for a year or more. Temporary older residents (living in their current residence for less than a year) and prospective research participants with communication difficulties, mental illness, and an inability to understand the contents of the study were excluded. The research protocol was approved by the Ethics Committees of the First Affiliated Hospital of Medical School at Shihezi University (KJ2020–017-01). Written informed consent was obtained from all study participants.

### Procedures

Data was collected from primary medical and health institutions in participants’ residential areas. All eligible participants completed a standardized paper questionnaire with the help of trained researchers. The questionnaire was designed based on the practical experience of researchers, expert evaluation, and a literature review. The questionnaire covered demographic characteristics, social networks, GAMR, health status, and ICOPE demand. Strict quality assurance and quality control procedures were followed to ensure the validity and reliability of the research data. All investigators and researchers had previously conducted field surveys and had experience of face-to-face interviews. The investigators and researchers were trained for a week on using instruments for data collection. Two supervisors, who were senior researchers with rich experience in investigation and were responsible for training, supervised the research implementation. All data were double entered in a database and then compared and corrected for errors.

## Measures

### Explained variable

The explained variables of this study were ICOPE demand, which integrates health and social needs and has some complexity. Due to the limitations of governmental resources, measuring ICOPE demand urgency should be given priority. Two indicators were selected to measure the ICOPE demand of older people in this study. One indicator was to determine the urgency of older people’s ICOPE demand by measuring ICOPE demand intensity. The other was to reflect the complexity of ICOPE demand of older people by measuring the number of service contents needed by them. The indicator can not only identify the priority of older people but also take into account their preferences. The ICOPE demand intensity was obtained from the question “Do you need medical and social care services?” scored on a 5-point scale (1 = not needed at all; 5 = very needed). The ICOPE demand diversity was measured based on the question “How many of the following integrated health and social care services do you need to be provided?” The question was adapted according to the Notice on the Issuance of Service Guidelines for Integrated Health and Social Care Institutions (Trial) (No. 24 [2019] of the general office of the National Health Commission) issued by the General Office of the National Health Commission, the General Office of the Ministry of Civil Affairs and the Office of the National Administration of Traditional Chinese Medicine. The ICOPE service contents included basic, medical, traditional Chinese medicine, nursing, rehabilitation, auxiliary, and psychological and spiritual support services. We obtained nine dichotomous items (i.e., yes/no answer) with a total score ranging from 0 to 9, where the score indicated the number of service demands. The ICOPE demand diversity indicator was divided into five categories of variables from“1 = no service demand” to “5 = ≥ four types of service demands” according to the number of service demands. The total score of ICOPE demand was 0–10 based on the two indicator scores of demand intensity and complexity. It was converted into five grade variables of low (0–2 points), relatively low (3–4 points), medium (5–6 points), relatively high (7–8 points), and high (9–10 points), which were coded as 1–5 points, respectively. The higher the score, the higher the demand intensity and complexity. Internal consistency was assessed using the Spearman-Brown coefficient (*ρ*), which was a less biased estimate of reliability for 2-item scale [37]. We found good internal reliability with *ρ* = 0.95. The assessment of content validity was done through nine experts in the field of health and social care service practice and research. The item content validity index (I-CVI) and scale content validity index (S-CVI) were implemented for content validity, according to a minimum preset a priori criterion value for I-CVI of 0.78 [38] and a criterion value for S-CVI of 0.83 [39]. The I-CVI ranged from 0.9 to 1.0 and the S-CVI of the scale was 0.88, indicating that the validity of the scale was good.

### Explanatory variable

GAMR was measured by the question “Distance from you to health care institutions?” with answer options of “very close,” “close,” “generally,” “far,” and “very far.” In this study, the options “very close,” “close,” and “generally” were considered to have higher GAMR than the remaining options.

Health status was measured using the self-rated health status and the number of chronic diseases, indicating subjective and objective health statuses, respectively.

Self-rated health status was measured on a 5-point Likert scale (1 = “very poor”, 2 = “poor”, 3 = “general”, 4 = “good”, 5 = “very good”) based on an individual’s self-perceived general health. This instrument has been widely used to evaluate and predict the health status of the older population [21, 22]. In this study, the options “very poor,” “poor,” and “generally” were considered to have poorer self-rated health status than the remaining options.

The question “Have you been told by a doctor that you have any of the diseases on this card?”, adapted from the questionnaire from the China Health and Retirement Longitudinal Study (CHARLS), assessed the number of chronic conditions. Chronic diseases included the 14 common chronic conditions (hypertension, dyslipidemia, diabetes, cancer, chronic lung disease, liver disease, heart disease, stroke, kidney disease, stomach or digestive system disease, emotional or mental problems, memory-related diseases, arthritis or rheumatism, and asthma) defined in the CHARLS. According to the number of chronic diseases, the indicator was divided into three categorical variables: “1 = no chronic disease,” “2 = one type of chronic disease,” and“3 = ≥ two types of chronic diseases.”

### Covariates

According to previous studies [5, 23, 24], demographic characteristics mainly included household registration, sex, age, education, monthly income, basic medical insurance, endowment insurance, spouse/partner, number of children, and number of friends. Supplementary Table S1 displays the details of relevant items.

Social network includes family social network resources owned by older people and their availability. The former was reflected by the number of spouses/partners, children, and friends. The latter was measured by the question “Can you get care and support from your spouse/children/friends when you need help?” with the answer options “yes” or “no.”

### Statistical analysis

The database was established by EpiData version 3.1, and statistical analysis was performed with STATA 16.1 (Stata Corporation, College Station, TX, USA). Data were presented as *N* (%) for categorical variables (i.e., sex, education) and as mean and standard deviation (*SD*) for continuous variables (i.e., ICOPE).

First, the differences in demographic characteristics, social networks, health status, and ICOPE demand between high and low GAMR groups were compared using the likelihood chi-square test for categorical variables and the two independent samples *t*-test for continuous variables.

Second, to reduce the influence of selection bias and confounding variables on the conclusions, propensity score matching (PSM) was used to estimate the influence of GAMR on ICOPE demand. PSM is a counterfactual estimation method that can be used to estimate the net effect on outcomes between treatment and control groups [40]. In this study, PSM was used to balance demographic characteristics of household registration (sex, age, educational level, monthly income, basic medical insurance, endowment insurance), and social network (spouse/partner, number of children, number of friends, spousal support, children support, and friends support) between two groups. Common support condition and balance tests were used to estimate the matching quality. The criterion for meeting the common support condition test was the distribution of propensity scores between the high and low GAMR groups as close as possible after matching. The criterion for meeting the balance test was that the characteristics between the two groups after matching were balanced with no significant difference. The nearest neighbor method with a 1:1 ratio (high and low GAMR groups) set in a caliper of 0.01 of the propensity score and an *SD* of 0.1 for the distance measure was applied to estimate the average treatment effect on the treated (ATT) of GAMR on ICOPE demand and compared the group differences among different health status. The 95% confidence interval (CI) of the ATT value was estimated by bootstrapping. The ATT values of nearest neighbor matching with a 1:4 ratio and radius (caliper) and kernel matching were used to analyze the robustness of the results. This study used a total of 2000 bootstrapping samples. All tests were 2-sided with a 5% significance level.

## Results

### Descriptive analysis

Of the 1200 potential research participants, 1121 participated in the survey, and 1043 met the inclusion criteria. Among the 1043 participants, 250 (24.0%) lived in areas with low GAMR, and 793 (76.0) lived in areas with high GAMR. The overall response and effective rates were 93.4 and 86.9%, respectively.

Table 1 summarizes the baseline characteristics of the study participants. Most (*n* = 814 [78%]) participants were from urban areas. Nearly half (*n* = 512 [49.1%]) were aged 70 to 79 years. Among participants, the majority were female (54.55%), had primary education or below (61.94%), had monthly income between 2001 and 4000 RMB (65.39%), and had employee medical insurance (77.37%) and endowment insurance (69.22%). Most participants had spouses (74.40%), two or more children (90.89%), and one or more friends (90.41%). Participants received the most support from their spouses and children when needed, with only 10.07% receiving support from their friends. For health status, 68.3% of participants rated their health status as “poor,” and 86.96% of them suffered from at least one chronic disease. The average score of all participants was 3.68 for ICOPE demand (*SD* = 0.78).

**Table 1:** Descriptive statistics of study variables

Variable	All samples (*N* = 1043)	High GAMR (*n* = 250)	Low GAMR (*n* = 793)	P
Household registration				0.586
Rural	229(22.0)	58(23.2)	171(21.6)	
City	814(78.0)	192(76.8)	622(78.4)	
Age (year)				0.925
60–69	355(34.0)	87(34.8)	268(33.8)	
70–79	512(49.1)	120(48.0)	392(49.4)	
≥ 80	176(16.9)	43(17.2)	133(16.8)	
Gender				0.282
Male	474(45.4)	121(48.4)	353(44.5)	
Female	569(54.6)	129(51.6)	440(55.5)	
Educational level				0.077
Primary School and below	646(61.9)	143(57.2)	503(63.4)	
Junior high school and above	397(38.1)	107(42.8)	290(36.6)	
Monthly income (RMB)				0.581
≥ 2000	159(15.2)	38(15.2)	121(15.3)	
2001–4000	682(65.4)	158(63.2)	524(66.1)	
> 4000	202(19.4)	54(21.6)	148(18.7)	
Basic medical insurance				0.552
Employee medical insurance	807(77.4)	190(76.0)	617(77.8)	
Resident medical insurance	236(22.6)	60(24.0)	176(22.2)	
Endowment insurance				0.040
No	321(30.8)	90(36.0)	231(29.1)	
Yes	722(69.2)	160(64.0)	562(70.9)	
Spouse status				0.406
No	267(25.6)	69(27.6)	198(25.0)	
Yes	776(77.4)	181(72.4)	595(75.0)	
Number of children				0.201
≤ 1	95(9.1)	25(10.0)	70(8.8)	
2	270(25.9)	54(21.6)	216(27.2)	
≥ 3	678(65.0)	171(68.4)	507(63.9)	
Number of friends				0.034
0	700(9.6)	79(7.6)	81(10.2)	
1–2	502(48.1)	138(55.2)	364(45.9)	
≥ 3	441(42.3)	93(37.2)	348(43.9)	
Spousal support				0.953
No	494(47.4)	118(74.2)	376(47.4)	
Yes	549(52.6)	132(52.8)	417(52.6)	
Children support				0.513
No	370(35.5)	93(37.2)	277(34.9)	
Yes	673(64.5)	157(62.8)	516(65.1)	
Friends support				0.445
No	938(89.9)	228(51.2)	710(89.5)	
Yes	105(10.1)	22(8.8)	83(10.5)	
Self-rated health				0.301
Good	328(31.4)	72(28.8)	256(32.3)	
Poor	715(68.6)	178(71.2)	537(67.7)	
Chronic disease				0.314
0	136(13.0)	38(15.2)	98(12.4)	
1	346(33.2)	87(34.8)	259(32.7)	
≥ 2	561(53.8)	125(50.0)	436(55.0)	
ICOPE demand	3.68(0.78)	3.51(0.05)	3.73(0.03)	< 0.001

Data are n (%) or mean (Standard errors)

*GAMR* geographic accessibility of medical resource, *ICOPE* integrated care for older people, *RMB* renminbi

Comparing the baseline characteristics between low and high GAMR groups, significant differences were found in education level, pension fund, and friends at the 10 and 5% levels, respectively. The ICOPE demand was significantly higher in the low GAMR group than in the high GAMR group (*p* < 0.01).

### Propensity score matching analysis

**Fig. 3:**
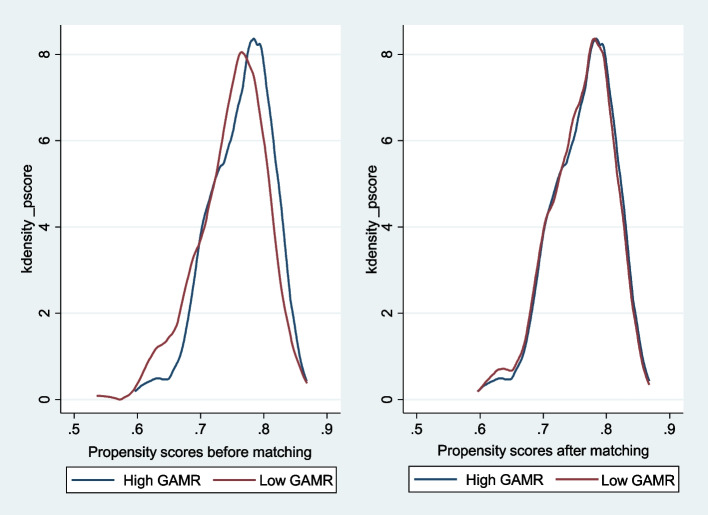
Distribution of the kernel density of the propensity scores of the ICOPE demand among older people. Note: GAMR: geographic accessibility of medical resource; ICOPE: integrated care for older people

Figure 3 and Table 2 show the propensity match. After matching, the kernel density curves of the high and low GAMR groups almost overlapped, and a large common support region below the curve overlap was seen. Thus, the data used in this study have suitable conditions for the common support domain, and maximum observations are within the range of the common value. The balancing tests of this study are shown in Table 3. Compared with before matching, after matching, the pseudo-*R*^2^ decreased from 0.013 to 0.002, the mean deviation and median deviation decreased, and the likelihood ratio indicated that the joint significance test for all variables was no longer significant. The results indicated that propensity score matching effectively balanced the distribution of covariates between the high and low GAMR groups and successfully reduced sample selection bias.

**Table 2:** Balance test

Sample	Ps *R*^2^	LR chi^2^	*P* > chi^2^	MeanBias	MedBias	β	R	%Var
Unmatched	0.013	14.44	0.343	6.0	5.0	27.5	0.91	14
Matched	0.002	3.76	0.993	2.3	2.0	9.7	1.24	14

**Table 3:** Average treatment effects of GAMR on ICOPE demand

Sample	Low GAMR	High GAMR	ATT	S.E.	T-stat	95%CL	P
ICOPE demand							
1 to 1 neighbor matching	3.731	3.461	0.270	0.067	3.98	0.123–0.418	0.000
1 to 4 neighbor matching	3.731	3.499	0.232	0.062	3.76	0.110–0.354	0.000
Radius matching	3.731	3.499	0.233	0.058	4.02	0.119–0.346	0.000
Kernel matching	3.731	3.502	0.230	0.056	4.07	0.119–0.341	0.000

Table 3 shows the average treatment effect of the impact of GAMR on ICOPE demand. After adjusting for covariates between high and low GAMR groups, ICOPE demand was 0.27 score units higher (1:1 match) higher in the low GAMR group than in the high GAMR group at a 1% significance level. The ATT values of nearest neighbor matching (1:4), radius matching method, and kernel matching method were similar to that of nearest neighbor matching (1:1), indicating that the results were robust.

Analyzing the impact of medical resource accessibility on ICOPE demand for older people with different health statuses showed that for both good and poor self-rated health status, ICOPE demand of the low GAMR group was significantly higher than the high GAMR group (ATT = 0.345, *p* < 0.05; ATT =0 .190, *p* < 0 .05). For older people with multimorbidity, the ICOPE demand in the low GAMR group was significantly higher than the high GAMR group (ATT = 0.318, *p* < 0.01) (Table 4).

**Table 4:** Average treatment effects of GAMR on medical resources on ICOPE demand

Sample	Low GAMR	High GAMR	ATT	S.E.	T-stat	95%*CL*	P
Self-rated health							
Good	3.579	3.234	0.345	0.113	3.04	0.076–0.614	0.012
Poor	3.819	3.629	0.190	0.091	2.07	0.011–0.369	0.038
Chronic disease							
0	3.590	3.131	0.459	0.187	2.45	−0.085–1.003	0.098
1	3.657	3.417	0.239	0.125	1.92	−0.031–0.510	0.083
≥ 2	3.806	3.488	0.318	0.108	2.94	0.119–0.517	0.002

## Discussion

This study reports for the first time the level of ICOPE demand that includes GAMR and health status among adults aged 60 years and older in Western China. First, our data indicate that the prevalence of chronic disease among older adults in the region exceeded the national level as a whole [41], but their self-rated health was adequate. Second, for the first time, the overall ICOPE demand of older people in the region was reported to be at a relatively high level (ICOPE demand score > 3). Third, our study findings show that 76% of older people faced low GAMR, and low GAMR increased ICOPE demand in older people.

Previous studies have reported that in China, 47.8–89.8% of older people aged 60 years and older suffered from chronic diseases, and 20–58.6% suffered from two or more chronic diseases [41–43]. We found that the prevalence of chronic diseases (87%) and multimorbidity (nearly 54%) in local older adults was high; however, more than 31% of older people considered themselves in good health, which was higher than previous studies [43] and the population without chronic diseases. Subjective health status is often closely related to declining intrinsic and functional capacity [20, 44]. Older people tend to consider themselves in good health when the decline in the aforementioned capacity does not exceed their range of adaptation [20, 45]. The gap between subjective and objective health status suggests that some older adults in this region can function effectively and independently perform daily chores and self-care activities despite experiencing physical diseases.

Differing from the previous service content perspective [9–11, 46, 47], in this study, we comprehensively measured ICOPE demand from the perspectives of demand intensity and complexity based on the Chinese ICOPE-related guidelines and the results indicated that older people had relatively strong demanded multiple ICOPE services. In China, most older adult services at home are undertaken by their families, and other older adult health services are provided by primary medical and health institutions. With the increasing ICOPE demand, primary medical and health institutions and families must collaborate to provide comprehensive and quality services to older people. However, more than three-quarters of older people reported that their families were remote from primary medical and health institutions. In particular, older people in the XPCC were also affected by poor infrastructure, such as transportation and communications [48], which further limited their access to primary healthcare services [49]. At the same time, with the advancement of urbanization, a large number of young and middle-aged workers went to economically developed cities to work, leading to an increasingly prominent rural family pension problem [50]. Evidence shows that with the increasing distance from home to the nearest primary medical and health institutions, the probability of losing self-care ability and abandoning treatment due to illness among older people increases significantly [28, 51, 52]. For older people living in remote rural areas, their ICOPE demands were met mainly by institutional health services and home care services without other alternatives such as telemedicine services and smart care services [53]. This causes them to have both high demand and high unmet demand problems. Although no significant differences in the health status of older people caused by GAMR were found, a significant increase in ICOPE demand caused by low GAMR was confirmed. We speculate that the health status of older people in the region may further deteriorate, which will spur demand, exacerbate the heterogeneity of ICOPE demand caused by geographic inequity of medical resources, and cause older adults to be trapped in a vicious circle.

Despite varied previous findings on the correlation between health status and ICOPE demand [8, 10, 15, 16], we captured the association between health and ICOPE demand from the perspective of geographic inequalities in health resources. First, although low GAMR has a significant positive effect on ICOPE demand in both older people with good and poor subjective health status, unsurprisingly, its positive impact on older people with good subjective health status seems more significant. Subjective health status is susceptible to the influence of the social environment [23, 28] when the medical resources access is blocked but is required to maintain health. Contrarily, the overall ICOPE demand level among older people with good subjective health status was relatively low in this study, which was lower than that of older people with poor subjective health status even after being affected by GAMR. Second, low GAMR significantly increased ICOPE demand for older people with multimorbidity. Multimorbidity is associated with decreased quality of life, impaired functional status, worsened physical and mental health, and increased mortality [54]. Therefore, older people with multimorbidity greatly depend on medical services in ICOPE. They have a stronger demand when homes are distant from primary medical and health institutions. These results suggest that geographic inequity in healthcare resources accelerates the demand release from older people with poor health and also stimulates demand growth from those who consider themselves healthy.

## Conclusions and recommendations

In summary, the subjective health status of older people in Western China is quite good. However, a high demand for ICOPE prevails due to the prevalence of chronic diseases and the geographical inequity of primary medical resources. This study argues that GAMR is a key factor in ICOPE demand growth in this region. Low GMAR accelerates demand release in both older people with comorbidity and those having both positive and negative perceptions self-perceptions of health.

Based on the results of the study, the administrative units of countries and regions with large areas and few people must create a geographic accessibility map of medical services. The administration must integrate the results of population aging and monitor health to optimize the spatial layout of primary medical resources to maximize accessibility. Additionally, for areas with inaccessible medical resources, information technology (IT) should be strengthened, and the spatiotemporal limitations of medical services should be reduced through telemedicine and intelligent medical services. Primary medical workers should be encouraged to increase on-site services to improve health services for older people.

### Study strengths

To the best of our knowledge, this is the first study to investigate ICOPE demand in the Western Chinese older adult population. The ICOPE demand comprehensively measures and considers demand intensity and complexity factors, overcoming the limitations of previous studies using only demand content measures. We excluded the interference of confounding factors to clearly present the relationship between GAMR and ICOPE demand, as well as the impact of health disparities. The findings can help health authorities optimize the layout of medical resources and promote health equity.

### Study limitations

Our study has a few limitations. First, the participants in this study were mainly from the western region (Xinjiang), which has a unique geographical environment. The extrapolation of the results must be supported by large samples from regions with different geographical environments. Second, due to the lack of data on spatial variables in the sample region, we only used the distance from older people’s homes to primary medical and health institutions as a measure of GAMR and did not consider the influence of transportation, which to a certain extent would have affected our comprehensive judgment of GAMR. However, based on the existing research [45, 46], this study forms the basis for evaluating the accessibility of medical resources. If conditions permit, these factors should be included in future studies to improve the precision of the measurement.

### Supplementary Information

**Supplementary Material 1.**

## Data Availability

The original contributions presented in the study are included in the article/supplementary material, further inquiries can be directed to the corresponding author.

## Abbreviations

ATT: Average treatment effect on the treated
CHARLS: China Health and Retirement Longitudinal Study
CI: Confidence interval
GAMR: Geographic accessibility of medical resource
ICOPE: Integrated care for older people
I-CVI: Item content validity index
IT: Information technology
*n*: Sample size
*N*: Population size
*p*: Probability (Spearman–Brown coefficient)
PSM: Propensity score matching
RMB: Renminbi
S-CVI: Scale content validity index
*SD*: Standardization deviation
WHO: World Health Organization
XPCC: Xinjiang Production and Construction Corps
