# Supplementary material for: Geographical accessibility of medical resources, health status, and demand of integrated care for older people: a cross-sectional survey from Western China
Source: BMC Geriatr. 2024 May 20;24:440. doi: 10.1186/s12877-024-04987-2 (PMC11103970; doi:10.1186/s12877-024-04987-2)
Supplement: Supplementary file 1 — Supplementary Material 1. [file 12877_2024_4987_MOESM1_ESM.docx]

**Supplementary Table S1:** Definition and descriptive statistics of various variables

| Variable | Variable description |
| --- | --- |
| **Explained variable** |  |
| ICOPE demand | Low = 1, lower = 2, generally = 3, higher = 4, high = 5 |
| **Explanatory variable** |  |
| GAMR | High = 0, low = 1 |
| Self-rated health status | Good = 0, [general](javascript:;) = 1, poor = 2 |
| Number of chronic disease | No chronic disease=1, 1 type of chronic diseases =2, ≥2 types of chronic diseases =3 |
| **Control variables** |  |
| **Demographic characteristics** |  |
| Household registration | Rural = 1, city = 2 |
| Gender | Male = 1, female = 2 |
| Age (year) | Continuous variable |
| Educational level | Primary School and below = 1, junior high school and above = 2 |
| Monthly income (yuan) | ≤ 2,000 = 1, 2,001–4,000 = 2, > 4,000 = 3 |
| Basic medical insurance | Employee medical insurance = 1, resident medical insurance = 2 |
| Endowment insurance | No = 0, yes = 1 |
| **Social network** |  |
| Spouse/partner | No = 0, yes = 1 |
| Number of children | $\leq$1 children=1, 2 children=2, ≥3 children=3 |
| Number of friends | No friend = 1, 1–2 friends = 2, 3–5 friends = 3, ≥ 6 friends = 4 |
| Spousal support | No = 0, yes = 1 |
| Children support | No = 0, yes = 1 |
| Friends support | No = 0, yes = 1 |
